# Reference values and factors associated with hand grip strength among older adults living in southeastern Poland

**DOI:** 10.1038/s41598-021-89408-9

**Published:** 2021-05-11

**Authors:** Agnieszka Wiśniowska-Szurlej, Agnieszka Ćwirlej-Sozańska, Justyna Kilian, Natalia Wołoszyn, Bernard Sozański, Anna Wilmowska-Pietruszyńska

**Affiliations:** 1 Institute of Health Sciences, Medical College of Rzeszow University, 35-310 Rzeszow, Poland; 2 Institute of Medicine, Medical College of Rzeszow University, 35-310 Rzeszow, Poland; 3 Faculty of Medicine, Lazarski University, 02-662 Warsaw, Poland

**Keywords:** Health care, Geriatrics

## Abstract

Handgrip strength (HGS) is used as a biomarker for the state of health of older people, but the number of research publications containing the normative values of HGS in older adult populations is limited. The aim of the study was to define reference values and factors associated with HGS in older adults living in southeastern Poland. A cross-sectional study including 405 participants aged 65 and older was conducted. Handgrip strength for the dominant hand was assessed by the average of three trials using a JAMAR dynamometer. The sample was categorized into the following age groups: 65–69 years, 70–74 years, 75–79 years, 80–84 years, 85 and over. The average HGS was 19.98 kg (16.91 kg for women and 26.19 kg for men). There was a decrease in handgrip strength across the age range in both sexes. The average handgrip strength of the older people was 17.97 kg (14.47 kg for women and 25.66 kg for men) for those aged 80–85 and 16.68 kg (13.51 kg for women and 21.77 kg for men) in the group over 85 years old. In both sexes, marital status was an independent factor associated with reduced handgrip strength. In conclusion, this study described, for the first time, handgrip strength values for the southeastern Polish population aged ≥ 65 years according to age and gender.

## Introduction

Handgrip strength (HGS) is a simple, fast and low-cost measure used in clinical practice, rehabilitation and public health research to determine musculoskeletal functions and evaluate frailty and disability^1^.

HGS is used as a biomarker for the state of health of older people^2^. It is directly related to the function of the upper limbs. Wang and Chen identified cut-offs for HGS necessary for grabbing and lifting heavy objects in older people (18.5 kg for women and 28.5 kg for men)^3^. Kim and Park found that HGS is correlated with upper limb disability^4^. Although we evaluate it using manual dynamometry, it allows us to identify not only upper limb muscle weakness but also lower limb strength and functioning^5^. It has been shown that poor performance standing up from a chair, walking and climbing up the stairs are associated with lower grip strength among older people^6^.

HGS is a simple method for stratifying the risk of death, regardless of other contributing factors, including physical activity, socioeconomic status and eating habits^7^. It plays an important role in the prognosis of clinical and surgical treatment and identifies patients with a lower chance of being independent after hospitalization^8^. Therefore, the availability of reliable and current HGS reference values for the older population is very important, and the simplicity of its measurement combined with its low cost makes it noteworthy for assessing the overall health of older people in both clinical applications and epidemiological studies.

The number of research publications containing the normative values of HGS in older adult populations is very limited. Reference values for older people in the German^9^, Swiss^10^ and Portuguese populations^11^ suggested that normative values may differ between countries due to varied physical, socioeconomic and cultural conditions^12^. In addition, normative data on HGS were reported by healthy elderly individuals living in society, which may not accurately reflect the true value of the parameter in the target population.

To the best of our knowledge, this is the first study in Poland presenting HGS reference values for older people. Therefore, the purpose of our study was to determine normative values of HGS and associated factors of hand grip strength for older adults living in southeastern Poland.

## Methods

### Study design

A cross-sectional study was carried out among a representative population of people aged 65 and older living in southeastern Poland (region of the Podkarpackie Voivodeship), including a total of 405 participants. The recruitment process included community-living older people and older people living in nursing homes to ensure a broad representative sample of the general older population. The sample size was composed of 4% older people living in nursing homes^13^; the proportion was described for the Polish older population. This study adheres to reporting guidelines for cross-sectional studies^14^.

Participants were included in the study based on the following inclusion criteria: age 65 years or older, normal cognitive status, Abbreviated Mental Test Score (AMTS) ≥ 6 points, a level of physical performance that enabled walking and provision of informed consent for participation in the study. The exclusion criteria were as follows: vestibular and neurological disorders, dizziness, paresis or deformities in the upper limbs and severe systemic diseases.

### Sample size

There were 349,124 people aged 65 and over in Podkarpackie Voivodeship^15^. The sample size was calculated using the Online Sample Size Calculator, assuming 95% confidence and a 5% margin of error. It was assumed that the minimum planned number of participants should be 400.

### Data collection and methods

Sociodemographic data, such as age, sex, marital status and education, were collected through interviews with study participants. The number of medications taken was also assessed, and body height and weight were measured.

**Hand grip strength (HGS)** was measured using a hand dynamometer (JAMAR PLUS + Digital Hand Dynamometer, Patterson Medical). The measurement was performed in a sitting position on a chair without armrests, with the feet of the examined person resting flat on the floor, arms set along the torso, the elbow flexed at 90 degrees, the forearm in a neutral position and the wrist in 0 degrees to 30 degrees extension following the procedure adopted by American Society of Hand Therapists^16^. The participant was instructed to clench the hand maximally and hold for 6 s. The procedure was performed three times for the dominant hand, with a one-minute rest between the tests. The average of three measurements (in kilograms) was recorded. The obtained result was classified as normal or low handgrip strength according to the criteria proposed by The European Working Group on Sarcopenia in Older People (EWGSOP)—the cut-off point for low handgrip strength was < 16 kg for women and < 27 kg for men^17^.

**The Activities of Daily Living and Instrumental Activities of Daily Living (ADL-IADL)** scale was used to assess the performance of basic and complex everyday activities^18,19^. ADL was assessed using six items based on the Katz Scale: bathing and showering, dressing, feeding, toilet hygiene, transferring—functional mobility and continence. IADL was assessed using six items based on the Lawton Scale: shopping, cleaning and maintaining the house, managing money, ability to use the telephone, taking medications and preparing meals. The summary ADL and IADL score ranges from low function, dependent, to high functioning, independent (ADL with a summary score from 0 to 6 and IADL with a summary score from 0 to 12).

**Timed Up and Go (TUG)** was used to assess mobility. Participants performed specific sequences of movements: getting up from the chair (height 41 cm with back support), walking a distance of three meters, turning around, walking back to the chair and sitting again^20^. The test was carried out three times, and the shortest time (in seconds) was used for the analysis.

**The Berg Balance Scale (BBS)** was used to assess static and dynamic body balance^21^. The scale included 14 tasks with gradually increasing difficulty, including changing body position, maintaining sitting position, maintaining standing position under visual control and without it, standing in a tandem position, standing on one leg, rotation around the axis, reaching forward, lifting objects from the floor, transferring, 360° turning and stepping.

### Ethics

In accordance with the Declaration of Helsinki, the participants were informed about the aim and course of the study and gave their informed consent to participate. The research project was approved by the Bioethics Committee of the University of Rzeszow.

### Statistical analysis

Data were analysed using statistical software R 3.6.3 (R Core Team, 2020) with the MKmisc (v1.6; Kohl, 2019) package (R Foundation for Statistical Computing, Vienna, Austria, URL: https://www.r-project.org/). For the analysis, the participants were stratified by gender. Descriptive characteristics were presented as a mean and standard deviations for continuous variables and a number and percent were used for categorical variables. Subject characteristics were compared using the Mann–Whitney U test (in the case of quantitative variables) and a chi-square test or Fisher test (in the case of categorical variables). The studied variables were not normally distributed, and Spearman’s rank correlation coefficient was used to investigate the relationship between quantitative variable data and HGS. Multivariate analysis of the simultaneous impact of many independent variables on HGS was performed by means of linear regression, and 95% confidence intervals were reported along with regression parameters. The significance level was set at p-value < 0.05.

## Results

In total, the study involved 405 older people, including 271 women and 134 men. The average HGS was 19.98 kg (16.91 kg for women and 26.19 kg for men). Low handgrip strength was reported among 50.18% women and 55.22% men. Height, body mass index, marital status, HGS and TUG were significantly different between women and men. The characteristics of the studied group are presented in Table 1.

**Table 1:** Sociodemographic characteristics of the study population.

	Women (n = 271)	Men (n = 134)	Total (n = 405)	p-value
	n (%) Mean (SD)			
**Age [years]**				
65–69	46 (16.97)	31 (23.13)	77 (19.01)	p = 0.137
70–74	46 (16.97)	24 (17.91)	70 (17.28)	
75–79	41 (15.13)	10 (7.46)	51 (12.59)	
80–84	101 (37.27)	46 (34.33)	147 (36.30)	
85 and more	37 (13.65)	23 (17.16)	60 (14.81)	
Body mass [kg]	69.27 (14.71)	76.35 (14.48)	71.61 (14.99)	p < 0.001 *
Height [cm]	157.02 (6.62)	169.08 (7.45)	161.01 (8.94)	p < 0.001 *
**BMI [kg/m**^**2**^**]**	28.03 (5.41)	26.73 (4.89)	27.6 (5.27)	p = 0.012 *
Underweight	2 (0.74)	2 (1.49)	4 (0.99)	p = 0.014 *
Normal body weight	82 (30.26)	54 (40.30)	136 (33.58)	
Overweight	94 (34.69)	51 (38.06)	145 (35.80)	
Obesity	93 (34.32)	27 (20.15)	120 (29.63)	
**Marital status**				
Married	50 (18.45)	59 (44.03)	109 (26.91)	p < 0.001 *
Widow / widower	157 (57.93)	44 (32.84)	201 (49.63)	
Divorced	9 (3.32)	13 (9.70)	22 (5.43)	
Single	55 (20.30)	18 (13.43)	73 (18.02)	
**Education**				
Basic	122 (45.02)	62 (46.27)	184 (45.43)	p = 0.742
Vocational	64 (23.62)	36 (26.87)	100 (24.69)	
Secondary	68 (25.09)	27 (20.15)	95 (23.46)	
Higher	17 (6.27)	9 (6.72)	26 (6.42)	
Number of drugs	5.02 (3.04)	5.6 (3.34)	5.21 (3.15)	p = 0.110
ADL	5.43 (0.87)	5.48 (0.81)	5.45 (0.85)	p = 0.713
IADL	9.36 (2.56)	9.23 (2.76)	9.32 (2.62)	p = 0.774
**Handgrip strength [kg]**	16.91 (6.95)	26.19 (9.66)	19.98 (9.06)	p < 0.001 *
Low grip strength cut off points ^a^	136 (50.18)	74 (55.22)	210 (51.85)	p = 0.396
TUG [s]	21.28 (15.21)	18.41 (14.12)	20.33 (14.9)	p = 0.043 *
BERG	38.34 (14.56)	39.25 (14.07)	38.64 (14.39)	p = 0.766

BMI: body mass index, ADL: basic activities of daily living, IADL: instrumental activities of daily living, TUG: Timed up and go test, BERG: Berg Balance Test, n: sample size, SD: standard deviation.

*Statistically significant (p < 0.05), ^a^ Low grip strength cut-off points for women < 16 kg, cut off points for men < 27 kg.

Among women, the 50th centile of handgrip strength ranged from 13.5 to 24.2 kg, and among men, it ranged from 22.2 to 29.2 kg. There was a decrease in HGS across the age range in both sexes. The average HGS of people aged 80–85 was 17.97 (14.47 kg for women and 25.66 kg for men), and in the group over 85 years old, it was 16.68 kg (13.51 kg for women and 21.77 kg for men). The mean values, with standard deviation and percentiles, of the HGS reference values for both sexes in 5-year intervals are shown in Table 2.

**Table 2:** Handgrip strength stratified by age groups, for both women and men.

Gender	Age	Mean (SD)	P5 (95%CI)	P10 (95%CI)	P25 (95%CI)	P50 (95%CI)	P75 (95%CI)	P90 (95%CI)	P95 (95%CI)
Women	65–69	22.38 (7.86)	9.05 (4.8–33.8)	11.90 (4.8–16.8)	16.85 (11–20.8)	24.20 (20.8–26.6)	28.73 (26.6–32.0)	31.20 (28.9–33.6)	33.05 (4.8–33.8)
	70–74	18.63 (6.97)	8.675 (7.4–34.8)	10.70 (7.5–12.6)	12.60 (10.1–14)	19.05 (14 .0–22.4)	24.23 (22.3–28.8)	27.80 (24.4–29.7)	29.025 (7.4–34.8)
	75–79	17.89 (6.76)	6.90 (5.4–29.0)	8.70 (5.4–11.9)	13.40 (8.7–15.6)	17.80 (14.3–21.1)	23.00 (19.5–27.9)	27.90 (24.9–29.0)	28.90 (5.4–29.0)
	80–85	14.47 (5.51)	6.30 (4.3–7.9)	7.90 (4.8–8.4)	10.10 (8.7–12)	14.00 (12.7–15.7)	18.50 (16.9–19.7)	21.40 (20.1–25.3)	23.70 (21.4–29.1)
	> 85	13.51 (4.19)	7.98 (3.7–22.1)	8.78 (3.7–9.8)	11.30 (8.9–12.6)	13.50 (10.2–14.5)	15.70 (13.9–20)	20.04 (17.0–22.1)	20.26 (3.7–22.1)
Men	65–69	29.96 (11.77)	11.05 (8.4–52.9)	14.20 (8.4–22.1)	22.55 (14.2–26.5)	29.20 (23.0–35.0)	39.90 (32.2–43.1)	43.10 (40.3–52.9)	48.45 (8.4–52.9)
	70–74	26.59 (11.98)	6.935 (6.4–47.4)	11.05 (6.4–47.4)	17.57 (9.4–27.9)	27.90 (19.6–32.5)	33.08 (27.9–43.9)	43.66 (6.4–47.4)	44.84 (6.4–47.4)
	75–79	26.10 (8.85)	13.39 (7.9–41.7)	18.88 (7.9–41.7)	22.55 (7.9–41.7)	26.45 (20.1–32.1)	30.78 (7.9–41.7)	33.06 (7.9–41.7)	37.38 (7.9–41.7)
	80–85	25.66 (6.42)	15.925 (12.9–43.7)	16.40 (15.3–22.0)	22.00 (16.3–23.9)	26.40 (23.9–27.6)	28.88 (27.6–35.1)	33.25 (28.9–36.6)	36.00 (12.9–43.7)
	> 85	21.77 (8.23)	7.21 (5.0–36.8)	10.74 (5.0–36.8)	17.70 (10.0–22.6)	22.20 (17.0–25.4)	26.85 (21.1–31.8)	31.72 (5.0–36.8)	32.79 (5.0–36.8)
Total	65–69	25.43 (10.26)	9.28 (4.8–12.6)	12.60 (8.4–16.8)	17.90 (13.6–22.1)	25.10 (22.8–27.6)	31.30 (29.1–34.5)	39.82 (33.1–43.1)	43.02 (39.5–52.9)
	70–74	21.36 (9.7)	7.82 (6.4–10.8)	10.55 (6.5–11.2)	13.10 (11.2–17.5)	20.95 (17.0–23.5)	27.68 (23.5–29.1)	32.73 (29.1–45.0)	41.98 (31.9–47.4)
	75–79	19.50 (7.84)	7.40 (5.4–41.7)	8.70 (6.2–13.0)	13.60 (10.4–17.6)	18.60 (17.1–22.0)	25.75 (21.1–27.9)	28.90 (26.7–32.1)	30.50 (5.4–41.7)
	80–85	17.97 (7.78)	6.86 (4.3–8.1)	8.32 (7.0–10.0)	12.15 (10.1–13.6)	17.10 (15.8–19.8)	23.10 (20.7–26.2)	28.74 (26.7–30.5)	30.57 (29.1–36.6)
	> 85	16.68 (7.23)	6.89 (3.7–9)	8.87 (5.0–10.0)	11.725 (9.8–13.9)	15.15 (13–17.4)	20.95 (17.1–22.6)	25.69 (22.5–32.9)	31.42 (23.5–36.8)

P: percentile, CI: confidence interval, SD: standard deviation.

HGS was negatively correlated with age in both women and men (women r = −0.395; p < 0.001 vs men r = −0.228; p = 0.008). There was a correlation between HGS and sociodemographic parameters, functional efficiency, mobility and body balance for women and men, while the number of medicines taken was only significant for women (r = 0.226; p < 0.001). Spearman’s rank correlation coefficients between HGS and sociodemographic parameters, functional status, mobility and body balance are shown in Table 3.

**Table 3:** Spearman’s rank correlation coefficients between handgrip strength and other variables.

Parameter	Women		Men	
	r	p	r	p
Age [years]	−0.395	p < 0.001 *	−0.228	p = 0.008 *
Body mass [kg]	0.337	p < 0.001 *	0.193	p = 0.025 *
Height [cm]	0.314	p < 0.001 *	0.205	p = 0.018 *
BMI [kg/m^2^]	0.233	p < 0.001 *	0.07	p = 0.418
Education	0.171	p = 0.005 *	0.366	p < 0.001 *
Number of drugs	−0.226	p < 0.001 *	−0.155	p = 0.073
ADL	0.395	p < 0.001 *	0.44	p < 0.001 *
IADL	0.448	p < 0.001 *	0.472	p < 0.001 *
TUG [s]	−0.615	p < 0.001 *	−0.522	p < 0.001 *
BERG	0.623	p < 0.001 *	0.566	p < 0.001 *

BMI: body mass index, ADL: basic activities of daily living, IADL: instrumental activities of daily living, TUG: Timed up and go test, BERG: Berg Balance Test.

Notes: * Statistically significant (p < 0.05).

Research has shown that marital status is an independent risk factor for HGS. Unmarried women had a 4.16 kg lower HGS, divorced women had a 3.92 kg HGS and widowed women had a 2.79 kg lower HGS than married women. The linear regression model also showed that among women, each subsequent point on the BERG scale increased the HGS value by 0.10 kg. For men, widower status was associated with a 4.28 kg lower HGS, and each point on the IADL scale raised the HGS by 0.75 kg. The multivariate analysis is shown in Table 4.

**Table 4:** Association between handgrip strength and other variables.

Variable		Regression parameter	Women			Regression parameter	Men		
			95%CI		p		95%CI		p
Age [years]	65–69	ref				ref			
	70–74	−1.361	−3.571	0.849	0.229	−3.194	−7.488	1.1	0.148
	75–79	−1.015	−3.369	1.34	0.399	−1.688	−7.162	3.787	0.547
	80–85	−2.202	−4.453	0.049	0.056	0.54	−3.416	4.496	0.79
	> 85	−0.423	−3.502	2.655	0.788	−0.588	−5.687	4.512	0.822
Body mass	[kg]	−0.295	−0.984	0.394	0.403	−0.929	−2.212	0.355	0.159
Height	[cm]	0.447	−0.158	1.051	0.149	0.948	−0.264	2.161	0.128
BMI	[kg/m2]	0.878	−0.826	2.583	0.313	2.772	−0.931	6.474	0.145
Marital status	Married	ref				ref			
	Widow/er	−2.792	−4.615	−0.97	0.003 *	−4.298	−7.32	−1.277	0.006 *
	Divorced	−3.927	−7.721	−0.133	0.044 *	−1.099	−5.991	3.793	0.661
	Single	−4.163	−6.322	−2.005	< 0.001 *	−3.168	−7.633	1.297	0.167
Education	Basic	ref				ref			
	Vocational	1.252	−0.567	3.072	0.179	0.715	−2.708	4.138	0.683
	Secondary	1.35	−0.299	2.999	0.11	2.963	−0.884	6.81	0.134
	Higher	1.295	−1.673	4.263	0.393	5.64	−0.062	11.342	0.055
Number of drugs		−0.169	−0.442	0.104	0.226	0.086	−0.407	0.579	0.732
ADL		0.822	−0.172	1.816	0.106	0.288	−2.027	2.602	0.808
IADL		0.171	−0.177	0.52	0.336	0.753	0.105	1.401	0.025 *
TUG	[s]	−0.043	−0.114	0.028	0.24	−0.109	−0.28	0.063	0.218
BERG		0.105	0.028	0.182	0.008 *	0.086	−0.075	0.247	0.295

BMI: body mass index, ADL: basic activities of daily living, IADL: instrumental activities of daily living, TUG: Timed up and go test, BERG: Berg Balance Test, CI: confidence interval.

*Statistically significant (p < 0.05).

## Discussion

Handgrip strength has been suggested as a biomarker of ageing^22^. It is a crucial and easily available indicator of current health status and predictor of future outcomes^23^. In this study, we established normative values and factors associated with hand grip strength in older adults living in southeastern Poland.

We showed that the average HGS for women was 16.91 kg, which is similar to the results from Portugal^11^ and much lower than that in Great Britain ^24^. The average HGS value for men in our study was 19.98 kg and was lower than that in Germany^9^ and Switzerland^10^. We also presented data for the oldest-old, among whom the HGS values were 13.99 kg for women and 23.71 kg for men, similar to the results from Spain^25^ and lower than those in Switzerland^10^. The differences in HGS according to age and sex between particular countries are likely due to genetic differences in muscle mass, lifestyle and health care of the older people^25^. Handgrip strength based on age helps to identify older people at risk of frailty and plan preventive health services to reduce the risk of their dependence and mobility limitations.

Low grip strength is a powerful predictor of poor patient outcomes, such as increased functional limitations, poor quality of life, longer hospital stays and death. To identify people with a clinically significant reduction in HGS, we used a cut-off value of hand grip less than 27 kg in men and 16 kg in women recommended by the EWGSOP^17^. Taking into account the indicated values, a reduction in muscle strength in approximately 50% of older women and men was shown. One of the reasons for such a high percentage of people with reduced muscle strength may be the insufficient level of physical activity among older people in Poland. In the group of individuals 45 to 65 years old, specific WHO recommendations were met by 4.2% of the population^26^, while in the group over 80 years old, they were met by only 0.6% of the population^27^. Additionally, state-provided geriatric care is insufficient and does not meet the real needs of the older population^28^. Therefore, an early assessment of HGS would allow the introduction of various forms of support, reducing future healthcare costs.

We have shown that HGS is negatively correlated with age for both women and men. Women and men showed a large decline in HGS in the 70–74 year group (3.75 kg for women vs 3.37 kg for men). Prevention of muscle strength deterioration in older people should be the focus of interventions at the end of the career and after retirement.

A positive association with HGS was demonstrated for height, weight, BMI, ability to perform basic and instrumental activities of daily living, mobility and body balance. Our findings are consistent with previous studies^10,29^.

In our study, we found that marital status was an independently associated factor affecting HGS among women and men. Older widowed, divorced or unmarried people are characterized by lower results for strength and functional fitness as well as a higher risk of mortality, with no difference between the sexes^30^. The incidence of older adults living alone during later life is on the rise in most of the world. The social isolation of lonely people increases functional limitations and reduces quality of life. This phenomenon causes greater demand for public interventions for older people^31^. It may therefore be concluded that older people living alone are particularly vulnerable and constitute a care-demanding subpopulation.

This study has some limitations. Firstly, despite the fact that the sample size determined in the study is relatively large, the generalizability is limited, because only older adults living in southeastern Poland were selected as subjects of the study. Thus, the results cannot be automatically extended to the entire Polish population. In addition, after a division according to gender and age, there are differences in the sizes of the subgroups. A small number of participants were studied in the group of men aged 75–59 years. Future research in a more representative older population with a prospective design is needed. Secondly, the participants of this study varied in terms of health status. The differences in values of the handgrip strength between the studies may be caused by the fact that other authors studied groups of older people with a specified degree of functionality^32^. Thirdly, future studies would need to take into account the incidence of cognitive impairment in the older adults to better understand its association with HGS.

## Conclusion

This study is the first time attempt to establish HGS values for the population of southeastern Polish inhabitants aged ≥ 65 years, according to age and sex. Any future research in this area may benefit from the obtained normative HGS values in order to plan and optimize preventive care and physical therapy for older patients.

## Data Availability

The datasets used and analysed during the current study are available from the corresponding author on reasonable request.
